# The Examination of Patients during Pregnancy

**Published:** 1908-05-23

**Authors:** 


### Obstetrics.

### THE EXAMINATION OF PATIENTS DURING PREGNANCY.

The desirability in her own interests of a prospective mother submitting to at least one pelvic examination during gestation is now not disputed by anyone, though many practitioners shrink from recommending it on account of the suspicion and reluctance with which the suggestion is still only too often regarded. But the advantages of the practice are so numerous that it is incumbent on the obstetrician engaged at least to point out that the very highest professional authority is unanimous on the question. Unfortunately there are women whose prejudices are so inaccessible to reason that they will promptly betake them to another practitioner if this advice is offered, and they may find him too pliant to endorse the obstetric prudence of his neighbour, who may incur considerable odium as a consequence of the attempt to do his duty.

The ideal course with patients sufficiently enlightened to submit to it is to make a complete pelvic examination about the end of the third month of pregnancy, and another which need only be abdominal about eight weeks before labour is expected. At the first examination, if the patient is a primigravida, the most important information required is as to the capacity of the pelvis. Broadly speaking, if it is not possible to reach the sacral promontory, and so to measure the diagonal conjugate, the pelvis may be regarded as unlikely to offer any obstacle to delivery: if it is possible to measure this dimension, the true conjugate may be deduced and any requisite measures concerted. Besides the estimation of the conjugate, there is also to be noted any abnormality of the pelvic viscera, such as retroversion of the uterus, fibroid of the uterus or cervix, and so on. In a multigravida the history of previous labours is more valuable than the exact estimation of the pelvic diameters, but this does not really abrogate the necessity for examination. For carcinoma of the cervix, though very rare in combination with pregnancy, is not unknown, and ovarian cysts, fibroids, and other obstacles to labour may have arisen since the last confinement.

It must be emphasised that this examination must be conducted with all due gentleness, for there are women in whom the slightest roughness will set up a miscarriage, just as there are others in whom it seems almost impossible to excite one. It is also to be remembered that the discovery of a retroverted uterus does not justify interference unless it is giving rise to symptoms; most cases restore themselves spontaneously, but it should be explained to the patient not to delay in seeking treatment if any symptoms of incarceration arise.

External measurements are unluckily of little use in detecting moderate or slight degrees of pelvic contraction. It is quite possible for the interspinous and intercristal measurements to be ten and eleven inches respectively, and even in some cases more, and yet for the conjugate to be half an inch or so less than the normal. It may safely be said that if the second of these external measurements exceeds the first there is considerable pelvic deformity; but it is not safe to conclude the opposite when the normal ratio, and even the normal measurements, are preserved. On the other hand, any deficiency of half an inch or more calls for careful investigation.

Nor is the diameter known as the external conjugate of great value; indeed, it is probably much more fallacious than those just discussed. An external conjugate which is less than 7¼ in. should arouse suspicion, and is often a sign of contracted true conjugate, but a good many cases of contraction have external conjugates exceeding this figure by a quarter, a half, and even three quarters of an inch. Nor is the amount of diminution much guide to the pelvic inadequacy: sometimes an external conjugate of seven inches is found with a true conjugate very nearly normal; at others with extreme contraction.

At this examination the condition of the circulatory, respiratory, and urinary systems should be investigated by inquiry as to the past personal history, supplemented by physical examination if necessary.

The object of the second examination towards the end of the seventh (calendar) month is chiefly, in those whose pelves are known or believed to be normal, the correction of breech presentations; and in those known or suspected to have contracted pelvis the estimation of the relative sizes of the inlet and the fœtal head. Twin pregnancies can also then be detected, and the necessary preparations ordered on a double scale. It is also possible to decide finally what shall be done in the case of tumours discovered earlier, and not removed by operation at the time. A small fibroid without symptoms does not demand operation during pregnancy unless there is reason to suppose that it will obstruct labour, and this it is often impossible to predict early in pregnancy. Later on it can be noticed whether the tumour has risen out of the pelvis to a position of safety, or whether, owing to its uterine relations or to adhesions, it is threatening to cause trouble.

Occasionally, too, a placenta prævia may be discovered before it gives rise to symptoms; although there is then no immediate treatment to be ordered, it is of some advantage to be able to warn the patient of impending difficulty and to advise her what she must do as soon as hæmorrhage commences.

There is one more point to mention. If there is any vaginal discharge noticed, other than the blandest leucorrhœa, it should be treated. This measure of prophylaxis is almost as important as Crédé's method, and much more often neglected. By the adoption of these precautionary examinations in every woman who is sensible enough to take the word of her obstetrician for their importance, we are consulting our own interests as well as those of the mother and her infant: on both grounds, but particularly on the latter, it is to be hoped that this growing practice may soon become universal.

